# Specialty grand challenge: how can we use integrative approaches to understand microbial community dynamics?

**DOI:** 10.3389/fsysb.2024.1432791

**Published:** 2024-06-17

**Authors:** Umer Zeeshan Ijaz, Aqsa Ameer, Farrukh Saleem, Farzana Gul, Ciara Keating, Sundus Javed

**Affiliations:** ^1^ Water and Environment Research Group, University of Glasgow, Mazumdar-Shaw Advanced Research Centre, Glasgow, United Kingdom; ^2^ Department of Molecular and Clinical Cancer Medicine, University of Liverpool, Liverpool, United Kingdom; ^3^ National University of Ireland, Galway, Ireland; ^4^ Department of Biosciences, COMSATS University Islamabad, Islamabad, Pakistan; ^5^ National Veterinary Laboratories, Ministry of National Food Security and Research, Islamabad, Pakistan; ^6^ Department of Engineering, Durham University, Durham, United Kingdom

**Keywords:** regression analysis, multivariate statistical analyses, integrative “omics”, microbiology, ecology

## Introduction

Microbiome studies have seen exponential growth since the advancement of next-generation sequencing (NGS) technologies (Qin, 2019)—albeit now old technologies! Sequencing approaches applied to mixed microbial populations involve either amplification of small genes such as the 16S rRNA gene (Johnson et al., 2019), or recovery of whole microbial genomes through shotgun metagenomics (Quince et al., 2017). The majority of observational and interventional studies are hypothesis-driven, with samples obtained either as case controls, spatially, or within temporal settings (Knight et al., 2018; Qian et al., 2020). Regardless of the methodology or study criteria, the analysis of the data through bioinformatics typically yields abundance/coverage tables that recover N (samples) x P (features) data within the chosen experimental or environmental context. Additional data (metadata) include parameters associated with the samples of interest. For environmental samples, these may include physicochemical parameters, and for human- or other host microbiome studies, additional data may include anthropometric measures and clinical data. Indeed, these data are essential to correlate treatments, conditions, or experimental variables with microbial community profiles.

The trend is increasingly geared toward collecting more and more metadata, such as the incorporation of metabolomics for metabolites (Bauermeister et al., 2021), metatranscriptomics for gene transcripts (Ojala et al., 2023), and metaproteomics for proteins (Armengaud, 2023). There are also commercial research services available such as Resistomap (https://www.resistomap.com/), which facilitates environmental monitoring of antibiotic resistance genes by offering a customizable target gene table using SmartChip qPCR. In host studies to unravel host-microbiome interactions, flow cytometry-based immunophenotyping is typically incorporated (Siebert et al., 2019). In clinical research, services such as Olink (https://olink.com) offer target platforms for protein biomarker analysis. This is based on a technology called Proximity Extension Assay, which uses labeled antibody pairs with DNA oligonucleotides that bind to the corresponding proteins in a sample. These oligonucleotides are then extended by DNA polymerase and are quantified through microfluidic qPCR. They offer different protein-associated panels/biomarkers with biological functions linked to cytokines, cardiovascular disease, immuno-oncology, neurology, oncology, inflammation, and several other biological processes. Recently, there has been a focus on the study of microbial ecosystems in their entirety, with the buzzword being the “exposome”, i.e., all the observable variations to which microbial communities are exposed (Gao et al., 2022; Gul et al., 2024). This plethora of additional data can then fill in the gaps of how the microbiome responds to the environment it is observed in. This will provide mechanistic insight into the function of microbial communities in a number of important contexts.

In this grand challenge review, we discuss numerous statistical approaches currently in use to find associations across multiple datasets sharing the same sampling space. Deducing discriminant features based on variations in the sampling space (case-control, spatial, temporal, etc.) and segregating them from features that remain fairly stable is a challenging issue. We also discuss challenges in their utility and where the gaps need to be filled.

Data that are not microbiome (sequence) data but provide further information about the microbiome samples are considered metadata. At the most basic level, metadata can be either categorical data (labeling of samples) or continuous data (for example, numerical data such as age, body mass index (BMI), anthropometric measures, and physicochemical parameters like pH and temperature). Additional metadata includes features recovered from other modalities, such as the metabolome (mainly continuous variables). While the goal is to capture as many perceived sources of variability within the confines of the environment in which the microbiome is observed, downstream statistical analyses become challenging and raise several questions:

• Of all the covariates that are captured, which ones should be included in the analyses?
• What about the confounders that are not captured?
• Do all covariates hold equal importance? Is there a way to rank them?
• If additional modalities generate a new set of features, where should the emphasis be in the downstream statistics once multiple feature tables are obtained? Finding discriminating features in the sampling space or finding correlating features across the datasets? Is there a trade-off?
• How do we translate an association between the covariate and an individual or subset of microbes into clinical or ecological relevance?
• Is linearity the best assumption to infer patterns of interest between the microbes and the covariates?
• How do we tackle heteroscedasticity and under-sampling particularly when there are more features than the number of samples P >> N?
• Which approach holds importance? Is a study-centric approach suitable where the emphasis is on features that remain stable or act discriminatory, or a taxa-centric approach, where given all observed variability, we can assess the ecological role of a particular microbe?
• Given the thousands of microbes that are detected, should we include all or some in the analyses? What is more important? Highly abundant microbes? Highly prevalent microbes? Highly interacting microbes?
• Is there any utility of rare biosphere in the analysis? How do we decide what is rare and is not a limitation of sequencing depth?
• How do we incorporate the inherent correlations that exist between samples, particularly in clinical studies, where a single subject has provided multiple samples?
• How can we assess the stability and complexity of microbial ecosystems in the wake of environmental perturbations?
• How can network reconstruction approaches be improved further? Given a network topology, how do we decide what the most influential species are?
• In spatial or temporal gradients, how do we compare datasets where the sampling time/space do not match?
• What is an appropriate normalization measure for different types of data?

### Grand challenge: how can we find a relationship between a specific variable in a sea of variability and noise?

To assess the relationship between a single continuous outcome data and all measured independent variables we typically apply regression modeling. Regression models describe the relationship between one or more independent variables and a dependent variable. One of the most commonly used models is the linear regression model. Regression coefficients generally referred to as β -coefficients, are associated with each continuous parameter and several categorical parameters. The categorical parameters are often *dummified* into a numerical representation, typically one less than the total number of factors observed in a parameter, through a procedure called one-hot-encoding. The variable that is excluded becomes a reference variable often denoted as REF. The formula for linear regression is $y_{i}=\beta_{0}+\beta_{1}x_{1,i}+\beta_{2}x_{2,i}+\beta_{3}x_{3,i}{+\beta }_{k}x_{k,i}+\varepsilon_{i}$ . The equation can also be written in matrix form as y=Xβ+ε . The signs of the β -coefficients give directionality with respect to the outcome y with the following interpretations: for continuous variables, a positive/negative coefficient is interpreted as “an increase/decrease in the covariate causes an increase/decrease in the outcome”; and for categorical variables, a positive/negative coefficient is interpreted as “as compared to the reference REF, the outcome Y is increasing/decreasing”. There are several extensions to the linear regression model, the most popular of which are discussed below.

The Generalized Linear Model (GLM) is a popular model (Xiao et al., 2018; Koh et al., 2019) in microbiome studies. In the GLM model, an outcome y is assumed to be generated from a certain distribution; relevant distributions include normal, binomial, Poisson, and negative binomial distributions, among others. The regression model is then defined as $g\left(\mu \right)=X\beta$ where μ is the conditional mean of the distribution, and $g\left(.\right)$ is the link function. The logistic regression is particularly important (i.e., the probability of an outcome with a specified variable), where the outcome is assumed to have a binomial distribution (the outcome variable takes values of 0 and 1) and the link function is the logit function $\ln\left(p/\left(1-p\right)\right)$ . However, we are interested in the log-binomial regression model which also assumes a binomial distribution for a binary outcome but uses a log link function lnp . Fitting a log-binomial regression model with binomial errors and a log link to binary outcome data thus makes it possible to estimate risk ratios by taking the exponential of the beta coefficients as $e^{\beta_{k}}$ , e.g., in (Firew et al., 2020). It should be noted that the risk ratio is the ratio of the probability of an outcome in an exposed group to the probability of an outcome in an unexposed group, and the log-binomial regression model was useful in the COVID-19 times to determine risks with occupational factors (Firew et al., 2020).

In many studies, the samples follow a case-control relationship, and therefore supplementing with a log-binomial regression model using all sources of variability fills in the gaps in our understanding. For outcome variables with more than two categories, *multinomial logistic regression* and *ordinal logistic regression* are recommended (Liang et al., 2020). For categorical outcomes, we obtain risk ratios from the models. On the other hand, for continuous outcomes, the typical strategy is to limit the number of variables (covariates) in the model, either through the *subset regression* approach applied to linear regression using R’s leaps package (Lumley et al., 2013) or the Least Absolute Shrinkage and Selection Operator (LASSO) approach using R’s glmnet package (Tay et al., 2023). In glmnet, “*The Relaxed LASSO*” is implemented which solves the following optimization problem, 1N∑i=1Nwilyi,β0+βTxi+λ1−αβ22/2+αβ1β0,βmin over a range of values of the tuning parameter λ , with $l\left(.\right)$ being the negative log-likelihood contribution to the observation i . The method incorporates an *elastic net* penalty controlled by α , a compromise between lasso regression ( α=1 ) and ridge regression ( α=0 ). The LASSO constraint forces some of the beta coefficients to go to zero, acting as a variable selection approach. If the study has supplementary ‘omics data as metadata, for example, targeted or untargeted metabolomics, then after appropriate normalization, e.g., probabilistic quotient normalization (Dieterle et al., 2006), these data can also be used in the LASSO regression. Furthermore, the approach is not just limited to continuous outcomes, as one can also use binary outcomes.

While the above models are mainly suitable for metadata, applying them to microbiome data is not straightforward. The read count data in 16S rRNA or metagenomic sequencing are typically summarized as a count table, and since the total sample read counts from an experiment, often referred to as the library size, are dependent on the sequencing technology, the absolute values are an artifact and present a challenge in how they are used in the regression models. Furthermore, depending on the depth of sequencing and the shape of the microbial community distribution (typically following a lognormal distribution), the table is highly sparse (50%–90% zero counts in the abundance table), often leading to overdispersion. These are the two main challenges that need to be addressed, i.e.,

• How do we effectively normalize the microbiome data when the samples do not have the same library size?
• In view of normalization procedures, how do we handle sparsity?

In the published literature, the above two questions are tackled in somewhat different ways, although there is no unifying framework. We list two recent regression approaches that address part of the problem with room for improvement.

**FIGURE 1:**
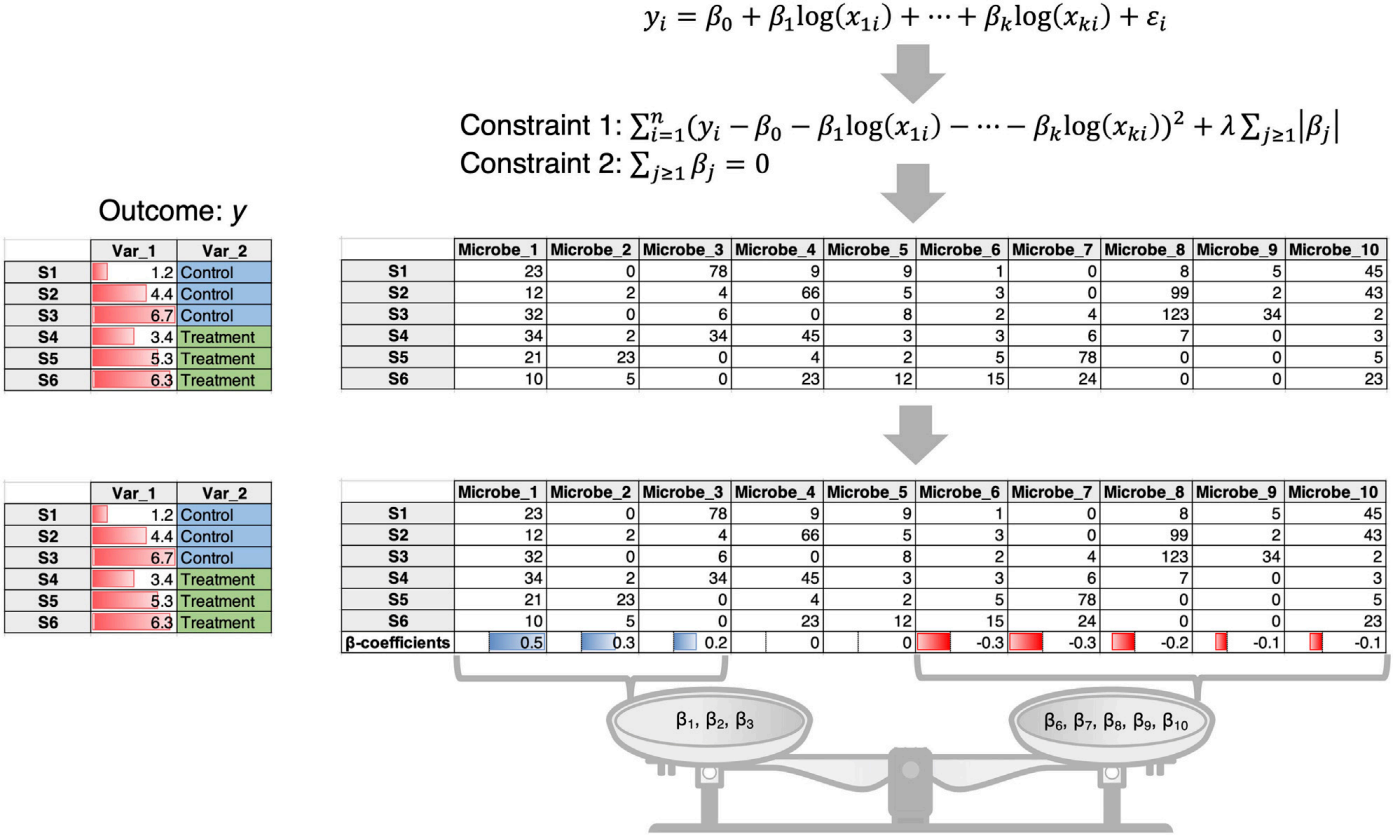
The CODA-LASSO approach extends the linear regression approach by taking log abundances of microbiome data and considering two additional constraints. Constraint 1 is the LASSO constraint that forces some of the beta coefficients to go to zero allowing for variable selection, while constraint 2 ensures that there are two subsets of beta coefficients of microbes, those that are positively associated with the outcome, and those that are negatively associated with the outcome.

**FIGURE 2:**
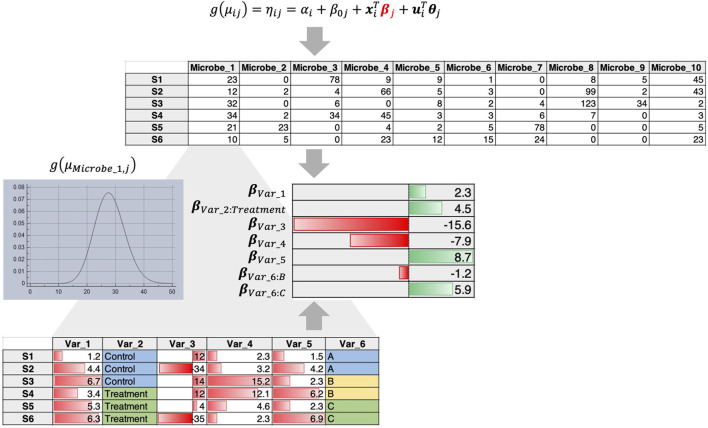
GLLVM procedure that fits a distribution for each microbe and regresses against all covariates to obtain beta coefficients that reveal positive or negative relationships.

1) In this case the microbial data are often taken to be compositional (i.e., the count table is converted to relative compositions constrained to 1). An example is the Compositional Decompositional Analysis (CODA)-LASSO approach (Calle et al., 2023) in which single binary/continuous outcome data from the meta table is regressed against the log abundances of microbes (Figure 1). While the approach offers variable selection by virtue of LASSO constraints, the challenging issue here is the log transform. The zero-count microbiome data cannot be log-transformed. A common practice is to add a pseudo-count, more commonly 1, 0.5 or even smaller values. There is no consensus on the appropriate choice of pseudo-count (Costea et al., 2014), and this remains an open problem to be solved despite recent attempts to address it (Hu et al., 2022).
2) In this case, abundance of individual microbes is regressed against covariates by fitting a distribution such as a Negative Binomial distribution that tackles overdispersion and sparsity. For example, using the *Generalized Linear Latent Variable Model* (GLLVM) approach (Niku et al., 2019) an extension of GLM, microbial abundances are regressed against all covariates including the latent variables (confounders that are not observed). The GLLVM approach (Figure 2) uses a link function g() similar to a GLM, fits a count distribution, and regresses against the covariates, where $\beta_{j}$ are the coefficients of the microbes associated with individual covariates. After estimating a 95% confidence interval for these coefficients, there are three possibilities for a given beta coefficient: the 95% confidence interval is all positive (an increase in the covariate causes an increase in the abundance of the given microbe); the 95% confidence interval is all negative (an increase in the covariate causes a decrease in the abundance of the given microbe); and where the 95% confidence crosses the zero threshold (the covariate is insignificant). For scenarios where the covariate is categorical in nature, it is dummified (converted to 0s and 1s) with one factor acting as a reference. The interpretation is similar to the explanation given above for continuous covariates, except that now the interpretation is with respect to the reference factor: the 95% confidence interval is all positive (as compared to the reference factor, there is an increase in the abundance of the given microbe); the 95% confidence interval is all negative (as compared to the reference factor, there is a decrease in the abundance of the given microbe); and the 95% confidence interval crosses the 0 boundary (the covariate is not significant). While $\beta_{j}$ are the coefficients associated with covariates $x_{i}$ , $\theta_{j}$ are the corresponding coefficients associated with latent variable $u_{i}$ . $\beta_{0j}$ are the intercepts and $\alpha_{i}$ are optional sample effects that can be chosen as either fixed effects or random effects. In addition, the residual covariance matrix $\Sigma =\Gamma \Gamma^{T}$ of the $\theta_{j}$ coefficients stores correlations between microbes where $\Gamma =\left[\theta_{1}\ldots \theta_{m}\right]$ for m latent variables. This residual covariance matrix can then indicate co-occurrence relationships between microbes that are not explained by the observed covariates. However, fitting GLLVM against exhaustive sources of variability when there are thousands of taxa, significantly more than the number of samples, is computationally challenging and impractical for larger datasets. Including all of them may not result in the convergence of the likelihood function. The open-ended questions are then: Within the framework of GLLVM, and other regression models in general, should we incorporate a subset of microbes? What should be the criteria for the inclusion of a microbe?

To limit the number of microbes in the regression model, the filtering criteria is often to ignore low abundance or low prevalence microbes. While this may have worked in several studies where the dominant role was played by the abundant or prevalent taxa, however, there is growing literature that emphasizes the importance of the rare biosphere (Lynch and Neufeld, 2015), which may have ecological, taxonomic, and functional potential. Furthermore, a recent study proposes that keystone interacting microbial species (Layeghifard et al., 2019) have far more clinical relevance than the abundant or prevalent ones. They have associated hubs from the network topology of interacting species with clinical covariates, demonstrating this approach to be better. However, more research is needed to identify keystone interacting species from microbiome datasets, particularly to formulate network-wide statistics that are not only robust against biases but also offer biological relevance. The *Integrated Value of Influence* (Salavaty et al., 2020) is a good starting point for this as a reasonable measure to identify keystone microbial species.

### Grand challenge: how can we unravel the mediating role of microbes?

While most microbiome studies focus on observing changes in microbes in a case-control setting emphasizing their differential abundances, there are trait or performance data (outcomes) that are not explicitly incorporated. Therefore, a few challenges that arise include:

• Can we identify microbes that play a mediating role between the treatments and an outcome of interest?
• What is the nature of mediation? Is it local or global? Is there no mediation at all?
• Can the mediating microbes be potential targets for the development of therapeutic or clinical interventions?

**FIGURE 3:**
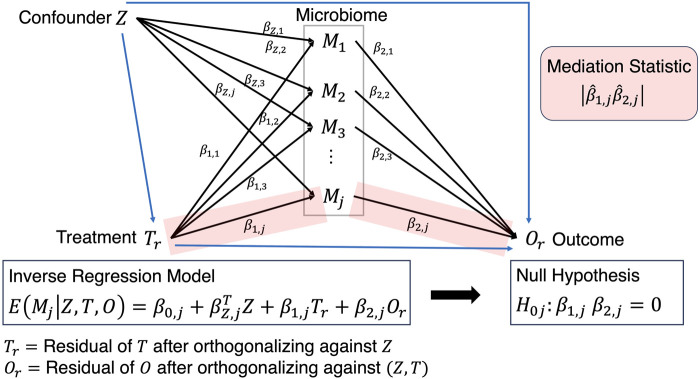
Mediation analysis where the path from *Treatment* to *Outcome* is established after solving the inverse regression problem and testing the corresponding beta coefficients that exist on that path.

Although still in its infancy, a recently proposed framework (Yue and Hu, 2022), simultaneously links Treatment T , microbial mediators $M=\left(M_{1},.,M_{J}\right)$ , outcome O , and confounding covariates Z . The classical model for multiple mediators (Van Der weele & Vansteelandt, 2013) is a double regression problem, where for a continuous outcome and J mediators, we have

$$E\left(M_{j}\left|Z\right.,T\right)=\alpha_{0,j}+\alpha_{Z,j}^{T}Z+\alpha_{1,j}T$$

$$E\left(O|Z,T,M_{1},\ldots ,M_{J}\right)=\theta_{0}+\theta_{Z}^{T}Z+\theta_{1}T+\sum_{j=1}^{J}\theta_{2,j}M_{j}$$

where the total mediation effect through $\left(M_{1},\ldots ,M_{J}\right)$ microbial mediators take the form $\sum_{j=1}^{J}\alpha_{1,j}\theta_{2,j}$ with $\alpha_{1,j}$ characterizing the effect of T on $M_{j}$ given Z , and $\theta_{2,j}$ characterizes the effect of $M_{j}$ on O given Z and T and all other $M_{j}$ s. Testing $\alpha_{1,j}\theta_{2,j}=0$ for an individual mediator achieves the purpose as the non-zero value indicates the contribution of $M_{j}$ to the overall mediation effect. $E\left(O|Z,T,M_{1},...,M_{J}\right)$ called forward outcome model is difficult to solve as there are more mediators than the number of samples, and therefore an inverse regression model is proposed (Figure 3) where orthogonalized versions of the residual of treatment T against Z defined as $T_{r}$ , and orthogonalized versions of the residual of O against $\left(Z,T\right)$ defined as $O_{r}$ are used, and the two equations are merged into a single equation,

$$E\left(M_{j}\left|Z\right.,T,O\right)=\beta_{0,j}+\beta_{Z,j}^{T}Z+\beta_{1,j}T_{r}+\beta_{2,j}O_{r}$$

such that $\beta_{1,j}$ corresponds to $\alpha_{1,j}$ , and $\beta_{2,j}$ corresponds to $\theta_{2,j}$ with the test becoming $\beta_{1,j}\beta_{2,j}=0$ for an individual mediator.

Further variations include causal mediation methods specifically designed to handle high-dimensional and compositional microbiome data (Wang et al., 2020). This rigorous Sparse Microbial Causal Mediation Model (Sparse MCMM) applies a linear log-contrast regression model and Dirichlet regression model to estimate the causal direct effect of treatment and microbiome mediation effects at both the community and individual taxon levels. PhyloMed (Hong et al., 2023) on the other hand discovers mediation signals by analyzing sub -compositions defined on the phylogenetic tree. While these mediation approaches offer a deeper understanding of the causal mediation effect of the microbiome and have a growing number of applications in microbiome studies, they are often context-dependent, and cater to those situations where a sizeable proportion of the microbial community changes. Where the changes are small or localized, the above mediation approaches do not appear to work well, and thus there is room for improvement.

### Grand challenge: how can we determine whether variables cause a shift in microbial community diversity?

Microbial diversity measures provide insight into the structure and dynamics of microbial communities. Differences within treatments (alpha diversity) and between treatments (beta diversity) can highlight responses in microbial populations to environmental or other conditions. Diversity measures themselves do not provide statistics, only trends. How can we statistically determine whether a variable is linked to patterns in diversity? First, we need to apply an algorithm to establish whether the variable (covariate) is causing a change in beta diversity between groups. In this regard, Permutational Multivariate Analysis of Variance (PERMANOVA) is a very useful tool as it employs any of the beta diversity dissimilarity metrics suitable for microbiome data (traditional ones include Bray-Curtis distance and UniFrac metrics) and can be applied to a wide range of complex models. PERMANOVA is a permutation test that uses an F test to assess whether the variances of two populations are equal by comparing groups of objects with the null hypothesis being that centroids and dispersions are equivalent. For each of the covariates, the test returns an R^2^ value which, if significant, is the percentage of variability in the microbiome explained by that covariate. As PERMANOVA is sensitive to the order of variables, it is often combined with a filtering process such as *Redundancy Analysis (RDA) with forward selection* (Vass et al., 2020). An alternate method is the *Fuzzy Set Ordination* (FSO) method (Roberts, 2009). Similar to PERMANOVA it uses dissimilarity metrics and metadata, but it is based on the principle of fuzzy set theory and reports correlation *R* as a quality-of-fit metric. Moving forward, two challenges need to be addressed:

1. All of these methods rely on distances that use all the measured microbiome count without incorporating individual covariances, i.e., the importance of individual microbial species is lost. Emerging approaches (Satten et al., 2017; Andries and Nikzad-Langerodi, 2022) do offer a bit of reprieve, but a concerted effort is required to develop this direction.
2. Another issue is that the majority of methods require multivariate uniformity of variability (homoscedasticity) and balanced sample sizes. In particular, PERMANOVA suffers from loss of power and type 1 error inflation (Alekseyenko, 2016). Therefore, there is a need to develop new robust methods that can ensure correct data analysis. The $W_{d}^{*}$ test (Hamidi et al., 2019) may be a good advancement in this direction, but there is a lack of a unified framework to tackle heteroscedasticity.

**FIGURE 4:**
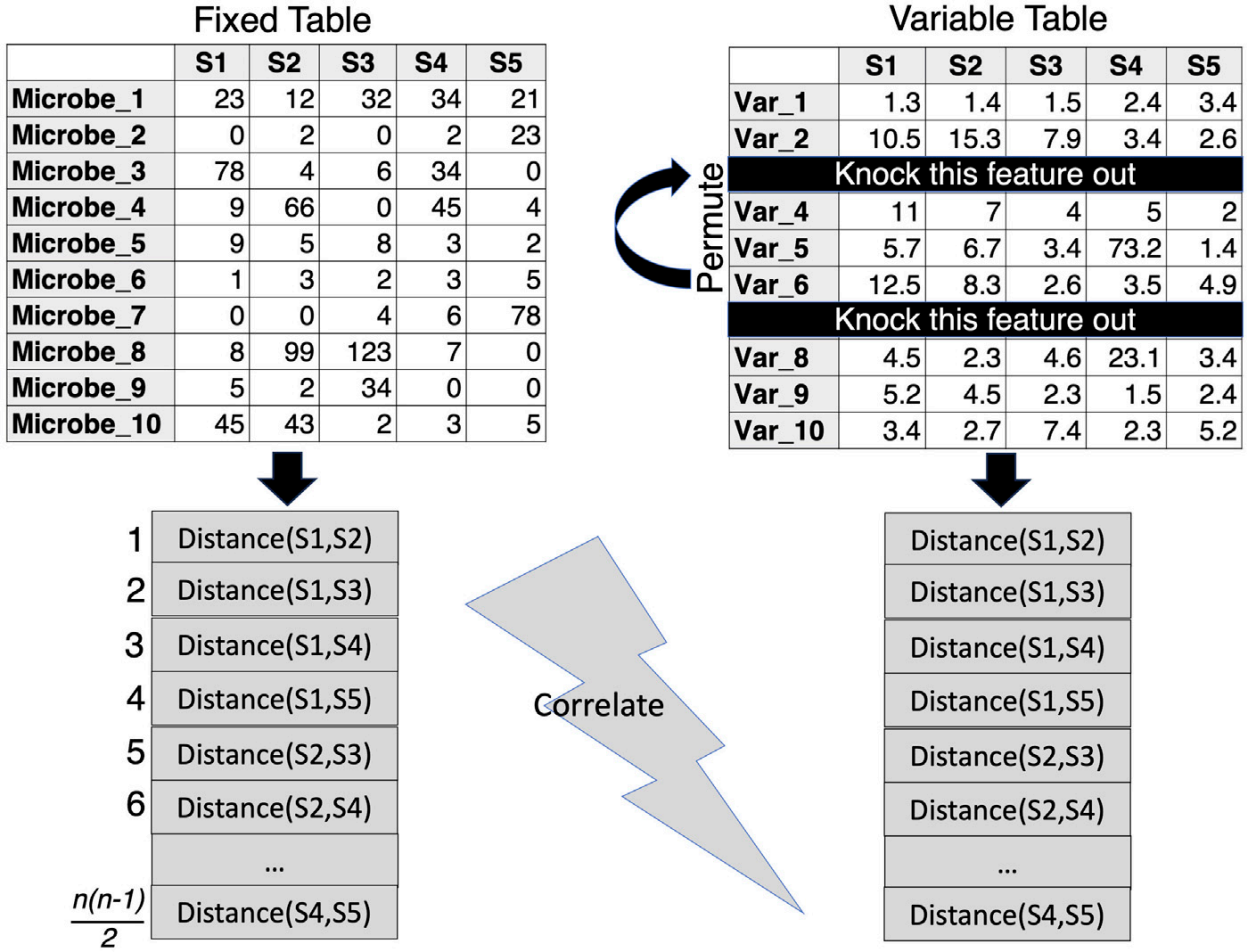
BIOENV approach, where the similarity distance is calculated for a fixed matrix (microbiome) and the features in the variable table are permuted to calculate a variable similarity distance in such a way that those subsets are retained where the correlation between the distances of the two matrices is optimized.

Perhaps an alternative to PERMANOVA could be to knock out unnecessary covariates through the approach by (Clarke and Ainsworth, 1993) which presents algorithms that allow for the comparison of beta diversity distances between two sets of data that have either samples or features in common. This approach facilitates the exploration of environmental variables (or clinical parameters) that best correlate with sample similarities in the biological community (microbiome). In the procedure (termed BIOENV), the similarity matrix of the community is fixed, while subsets of the environmental variables are used in the calculation of the environmental similarity matrix. A correlation coefficient is then calculated between the two matrices and the best subset of environmental variables can then be identified and further subjected to a permutation test to determine significance. R’s vegan package (Dixon, 2003) implements the bioenv() function, where the similarity matrix of environmental data is assumed to be based on normalized Euclidean distances (Figure 4). This makes sense with environmental data where one normalizes the data to remove the effect of differing scales between parameters. For the microbiome, the Bray-Curtis Similarity Index is commonly used due to its non-parametric nature.

### Grand challenge: how can we visualize the relationship between covariates and microbial diversity patterns?

**FIGURE 5:**
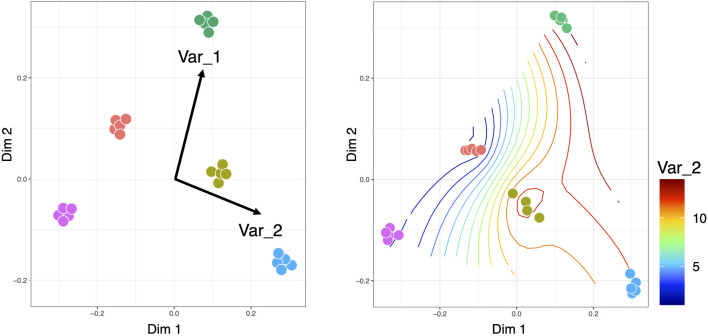
Constrained ordinations, where the left side visualization is obtained for CCA/RDA with the length of the arrows pointing to the direction of increase of the continuous covariates, while the right side is the smooth contour of a continuous covariate on the reduced ordination space. It should be noted that for CCA/RDA categorical variables can also be used where we get separate vectors for each factor of a categorical variable.

Constrained ordination approaches can be used to visualize the variation in microbial communities that can be explained by external environmental variables or constraints. There are different types of constrained ordination approaches such as Canonical Correspondence Analysis (CCA) and Redundancy Analysis (RDA) (Legendre and Legendre, 2012) (Figure 5). In the case of CCA, a Chi-squared transformed microbial abundance table is subjected to weighted linear regression on the constraining variable. The fitted values are then subjected to correspondence analysis using Singular Value Decomposition (SVD). RDA on the other hand uses ordinary unweighted linear regression and unweighted SVD. Additionally, there is a distance-based Redundancy Analysis (dbRDA) that allows non-Euclidean dissimilarity indices such as the Bray-Curtis distance. There are two versions of dbRDA implemented in R’s vegan package: the capscale() function based on (Anderson and Legendre, 1999); and the dbrda() function based on (McArdle and Anderson, 2001). The two methods differ in how dissimilarities are handled but they essentially do the same thing. To facilitate stepwise model building for constrained ordination methods, one can use the ordistep() function (Blanchet et al., 2008) from R’s Vegan package, which can perform forward, backward, and stepwise model selection using a permutation test. Alternatively, the ordisurf() function from R’s Vegan package can fit smooth surfaces using penalized splines (Wood, 2003) in the *Generalized Additive Model* (GAM). The method uses a single continuous metadata and regresses it against the smooth values of the scores obtained from any of the ordination techniques such as Non-Metric Distance Scaling (NMDS) or Principal Coordinate Analysis (PCoA). Nevertheless, there are challenges that need to be addressed in constrained ordination approaches. One of the shortcomings is the assumption of linearity in approximating the response of microbial species to environmental gradients (which typically follow a log-linear relationship). Although (Makarenkov and Legendre, 2002) provided a non-linear approach based on polynomial regression, more research is required in this area. Another problem is that CCA does not work well when there is a large variability in the library sizes of microbial samples (typical of metagenomics datasets), often leading to inflated Type 1 errors (Ter Braak and Te Beest, 2022). Further research is needed on the choice of test statistics associated with CCA to address this issue (Ter Braak, 2022).

### Grand challenge: how can we assess stability and complexity in microbiome studies?

Many studies today are concerned with assessing the stability of an observed microbial ecosystem against environmental perturbations (Mills et al., 2023) and whether the structure of the microbiome offers some sort of resilience. Typically, it is assumed that higher taxonomic diversity leads to higher functional redundancy which may provide an advantage when individual taxa are displaced or knocked out (the author’s paper above suggests otherwise). The challenge here is to come up with easy-to-use metrics to assess how stable an ecosystem is. Stability can be defined in terms of functional stability. For example, in (Eng and Borenstein, 2018a), artificial perturbations in taxonomic composition are created, and function $f=\frac{1}{e^{a}}t^{b}$ is fitted between the taxonomic difference *t* (using Weighted UniFrac) and the functional difference *f* (cosine dissimilarity between the original and perturbed functional profiles) of these perturbations to give *Attenuation a* and *Buffering b* coefficients. On a Buffering-Attenuation plot, these authors have compared different environments, showing gut communities to be more robust while vaginal communities to be unstable. However, to apply this procedure, the predicted functional profiles of individual observed taxa need to be known in advance which may be impractical for 16S rRNA studies unless there is an improvement in the database development of metabolic prediction software such as PICRUSt2 (Douglas et al., 2020).

Other approaches stem from May’s stability theory (May 1972), which states that the stability of n interacting microbial species is determined by the interacting community matrix M (also called the “adjacency matrix” obtained by network inference). The complexity is defined as $\alpha^{2}nC$ , where $\alpha^{2}$ and C are the variance and density of the non-zero off-diagonal components of M . With the scaled diagonal components as −1, the ecosystem is stable as long as it satisfies the stability criterion: $\alpha \sqrt{nC}<1$ . There are issues associated with the application of this theory to microbial interaction networks:

a) It is not possible to accurately reconstruct an interaction network from abundance data, as this would require high-quality time series data, and well-designed control experiments.
b) The current network reconstruction approaches (e.g., SPIEC-EASI (Kurtz et al., 2015), SparCC (Friedman and Alm, 2012), Phi statistics (Lovell et al., 2015), Probabilistic co-occurrence (Veech, 2013), MENA (Deng et al., 2012), etc.) that infer the community matrix M as a co-occurrence network are not really useful because they do not encode causal relationships.

The second problem can be addressed by estimating the effective connectance $D^{2}$ after fitting a regression model to samples that overlap in terms of the species they share and the sample dissimilarities (Yonatan et al., 2022). This avoids the need to infer a co-occurrence relationship explicitly, leading to $D^{2}$ serving as a proxy for stability. $D^{2}$ is then obtained by the slope of the regression fitted to the dissimilarity-overlap plot on the 25% top overlap values for the paired-wise dissimilarity/overlap values for N samples in a given category from a total of $N\left(N-1\right)/2$ paired-wise values. However, this approach requires at least 35 biological replicates which may be impractical for most studies.

For temporal datasets, a community-level measure of stability can be the *Local Contribution to Beta Diversity* (LCBD) measure (Legendre and De Cáceres, 2013). Any deviation from the mean LCBD can serve as a means to assess the stability of the system (Ijaz et al., 2018). Another advantage is that LCBD is a unidimensional measure that can be used in the regression approaches discussed previously and can be studied in the presence of covariates.

**FIGURE 6:**
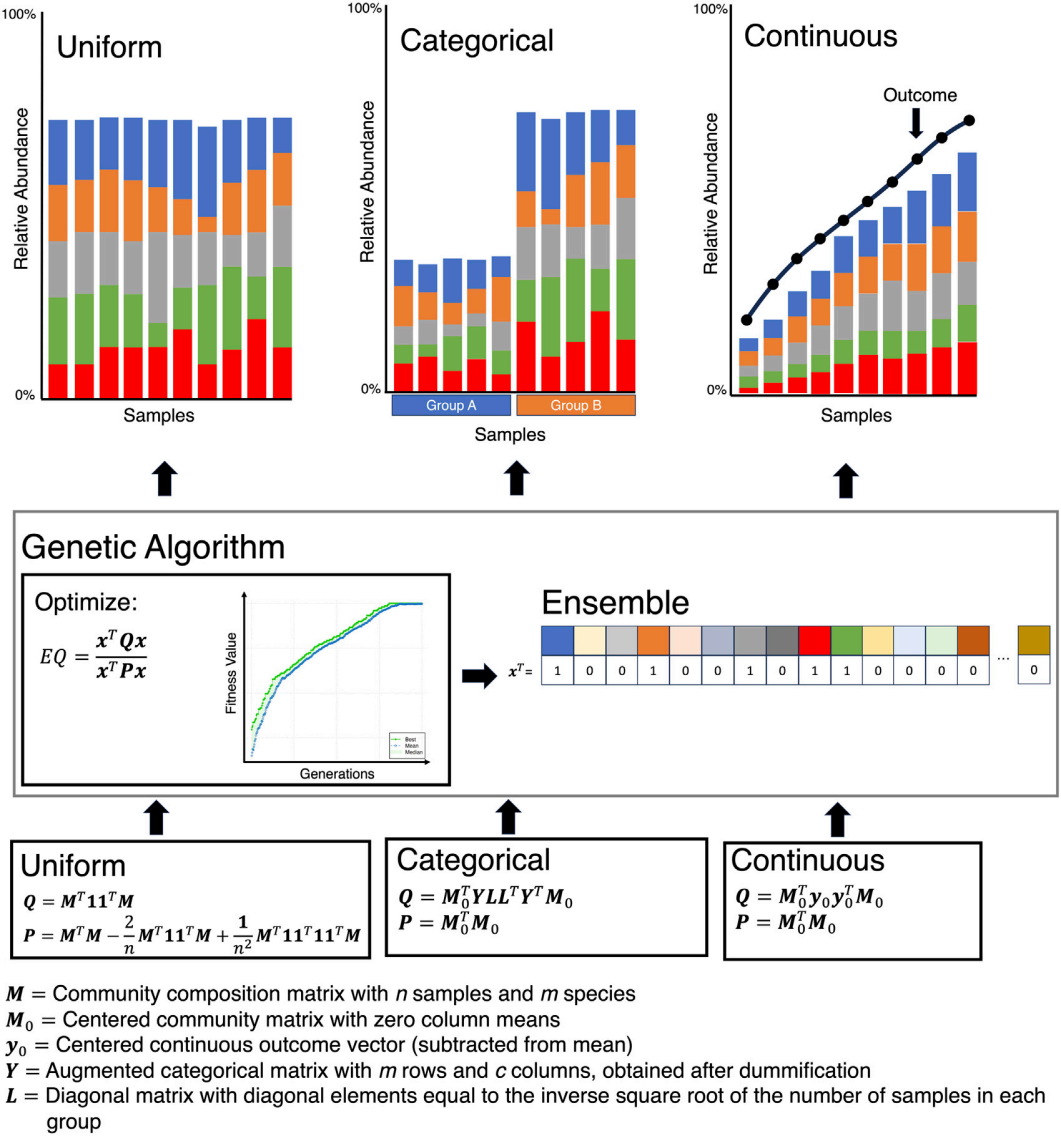
The Ensemble Quotient optimization algorithm works by finding an ensemble (a subset of microbes) that either remains stable (Uniform), has a stratified response (Categorical), or correlates with a covariate of interest (Continuous).

Moving on from community-level stability metrics to the identification of subcommunities that remain stable is another challenging issue. Not much work has been done in this direction, and it is still in its infancy. An important development in finding a minimal subset of microbes that either remain stable or change with respect to a continuous covariate of interest, is the Ensemble Quotient Optimization (EQO) approach by (Shan et al., 2023) (Figure 6). The approach uses a relative abundance table, called the community matrix M (*m* microbes over *n* samples), where the goal is to obtain a vector $x\in {\left(0,1\right)}^{P}$ where the *i*th position in the vector is either 0 or 1, i.e., a subset of microbes where values of 1 belong to an ensemble that we are interested in recovering. This ensemble is recovered in the context of a phenotype/predictor variable y by optimizing an *Ensemble Quotient* $EQ=\frac{x^{T}Qx}{x^{T}Px}$ , through a genetic algorithm (an optimization algorithm), where P and Q are algebraic transformations of the community matrix that capture the covariance between microbes, and the covariance between microbes and y . The choice of y dictates which ensemble one can recover, and it can be used in three cases: a) If the interest lies in an ensemble of microbes *that remains stable* for a set of samples, then y is considered uniform, i.e., consisting of 1s; b) If the interest lies in an ensemble of species whose cumulative abundance correlates with a continuous physico-chemical parameter y , then the algorithm is optimized with a centered community matrix $M_{0}$ , and a centered continuous parameter $y_{0}$ ; and c) if the interest lies in an ensemble of species whose cumulative abundance is stratified across different categories, then an augmented Y matrix is considered that captures the categorical information as 1s or 0s after applying dummification, and uses $M_{0}$ **.** In the context of temporal data, case (a) can be used to see which subset of microbes does not change over the whole time span (quality of fit is returned as the Coefficient of Variation CV), while case (b) can be used to see which subset of microbes has a relationship with the performance parameters (quality of fit is returned as the correlation coefficient between the continuous outcome and the cumulative abundance of the ensemble). In case (c) of a stratified response, the quality of fit is established by the Coefficient of Determination CD. To optimize the EQ to obtain x , the genetic algorithm optimization code is located at https://github.com/Xiaoyu2425/Ensemble-Quotient-Optimization.

In summary, the following questions need to be addressed:

• How can we construct microbial networks that can accurately capture microbial interactions including causality, and that too with reduced sample numbers?
• Can we construct easy-to-use metrics that can describe the complexity and stability of an ecosystem at both temporal and spatial scales, and that can seamlessly integrate with metadata?
• How can we numerically assess the resilience of an observed microbial community?
• What is the role of a stable subcommunity in the functioning of a microbial ecosystem?
• While the existing literature focuses on taxonomic stability, how can we recover stable functions from microbial ecosystems and relate them to the covariates (metadata)?

### Grand challenge: should more emphasis be given to taxa-centric approaches as opposed to study-centric ones?

The taxa-centric approach differs from the study-centric approach in that the emphasis is on elucidating how a particular microbe behaves in a variety of environments. In ecology, one of the important methods is to assess which niches microbes occupy, and whether there is a degree of overlap between them. For such an assessment, it is important to consider all possible sets of environments (dictated by biotic or abiotic variation), with the total number of environments serving as a parameter in the model. To identify the roles of microbes in the context of these environments, R’s MicroNiche package (Finn et al., 2020) is useful. It facilitates the identification of *generalist* (which should exist in the majority of the environments) and *specialist* (which should exist in some environments) microbial species in addition to the environment-dependent positive/negative association of microbial species with the continuous covariates observed in the data. Why is this important? There is growing literature suggesting that generalist and specialist species impact the microbial community dynamics differently (Sriswasdi et al., 2017), with generalists in particular playing a key role in maintaining taxonomic diversity. In fact, an author’s recent work (Mills, 2023) suggests that different microbial communities are disproportionately impacted by environmental disturbances, and stable environments lead to the proliferation of generalist species. Therefore, there is a need to consider the ecological roles of microbial species, and to distinguish between different categories of microbial species when studying a microbial ecosystem (Xu et al., 2022). How to identify distinct roles remains a challenge. Here, we discuss the recent taxa-centric approaches.

Before making distinctions in the MicroNiche framework, as a pre-processing step, microbes are first selected using the limit of quantification (LOQ) approach. Briefly, LOQ filters out microbes that fall below a “decision boundary”, calculated from the distribution of microbes with 95% confidence that these microbes will fall within a null distribution where the mean microbial abundance is zero. To calculate the standard deviation of the null distribution, the lognormal rank distribution of the microbes with the dataset is fitted with $S\left(R\right)=S_{0}e^{-a^{2}R^{2}}$ where the log abundance of the microbe S at rank R is dependent on the coefficient a and rank R calculated as $a=\sqrt{\frac{\ln S_{0}}{S_{m}}/R^{2}}$ where $S_{m}$ is the lowest taxon abundance of S . To calculate the LOQ, we fit the above log-normal model to the data, and the LOQ is then determined as the overlap between the null hypothesis (i.e., a microbe’s mean abundance is zero) and where the microbe falls within 1 standard deviation of the above model. After filtering out the microbes, we then calculated the niche breadth as Levins’ $B_{N}=\frac{1}{R}\sum_{i=1}p_{i}^{2}$ , where $p_{i}$ is the proportional abundance of a microbe in the i -th environment, with the total number of environments being R . If $B_{N}$ approaches 1 for a given microbe, then it is considered a “generalist”, while if it approaches 1/R , then it can be considered a “specialist”. To derive the *p*-value for Levins’ $B_{N}$ i.e., whether we can call a microbe a generalist or a specialist with a high degree of certainty, a null modeling approach is used, where a random normal distribution of 999 possible niche breadths are produced for a microbe, and allows a *p*-value to be assigned depending on whether a microbe’s $B_{N}$ is greater or lower than the mean of the null model. As per the author’s recommendation, after applying null modeling, the fifth Quantile and 95th Quantile are obtained to tag the microbes as specialists if its $B_{N}$ < fifth Quantile, and generalists if its $B_{N}$ > 95th Quantile. Those that fell in the inter-range were tagged as undecided.

**FIGURE 7:**
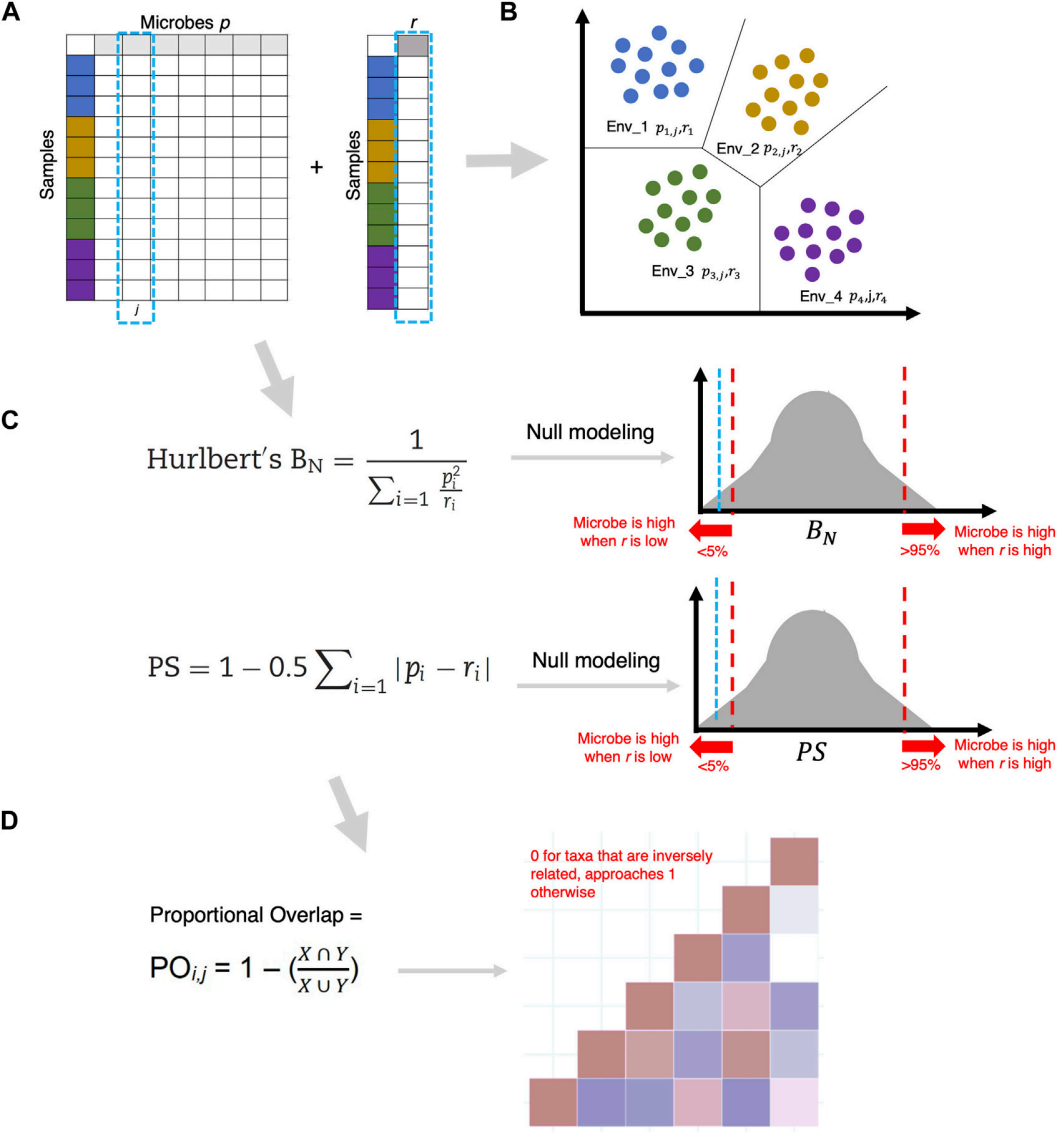
**(A)** Given $p_{i}$ , the proportional abundance of a microbe in the i -th environment, $r_{i}$ an environmental property (continuous covariate) is associated with each environment. **(B)** In the total number of environments being R (stratification based on some commonality among samples), we can assume for $p_{i}$ , and $r_{i}$ to be fairly stable within the environment and vary more across the environments. **(C)** Hurlbert’s $B_{N}$ and Feinsinger’s PS statistic indicates a negative or positive relationship between a microbe and an environmental property. A null modeling procedure is considered by generating a random normal distribution with 999 possible estimates, and by labeling a microbe as “negative” if the metric <fifth Quantile, and “positive”, if the metric >95th Quantile. **(D)** For Proportional Overlap $PO_{i,j}$ , *X* (for microbe *i*) and *Y* (for microbe *j*) are Feinsinger’s PS.

In the second step, the overlap of these specialist or generalist microbes is calculated using Levins’ Overlap formula $LO_{i,j}=\frac{\sum_{i,j=1}\left(p_{ir}\right)\left(p_{jr}\right)}{\sum_{i=1}\left(p_{ir}^{2}\right)}$ , where $p_{i}$ is the proportional abundance of the microbe i in the r -th environment, and $p_{j}$ is the abundance of the microbe j in the r -th environment. In addition to Levins’ $B_{N}$ , one can calculate Hurlbert’s $B_{N}$ , where an additional covariate $r_{i}$ observed for the environment i is incorporated in the formula (Figure 7). The model yields a value between 0 and 1 for each microbe and corresponding covariate, indicating whether there is an inverse (∼0) or a positive relationship (∼1), with 0.5 indicating no relationship to the covariate. To determine positive and negative relationships (potentially symbiosis and antagonism) between the microbes, Proportional Overlap $PO_{i,j}$ is used. The Proportional Overlap $PO_{i,j}$ is a Jaccard similarity coefficient that approaches 0 for microbe pairs that are inversely related to each other and approaches 1 for microbe pairs that are positively related to each other. Similar to the above approach is the development of the *Social Niche Breadth* score (von Meijenfeldt et al., 2023) which revealed across ∼22,000 environments that social generalists have a diverse pan-genome, are mainly opportunistic, and dominate local communities. Social specialists, on the other hand, exhibit mixed behavior, i.e., they are stable but low in abundance, and their genome sizes change with the diversity of their environments.

**FIGURE 8:**
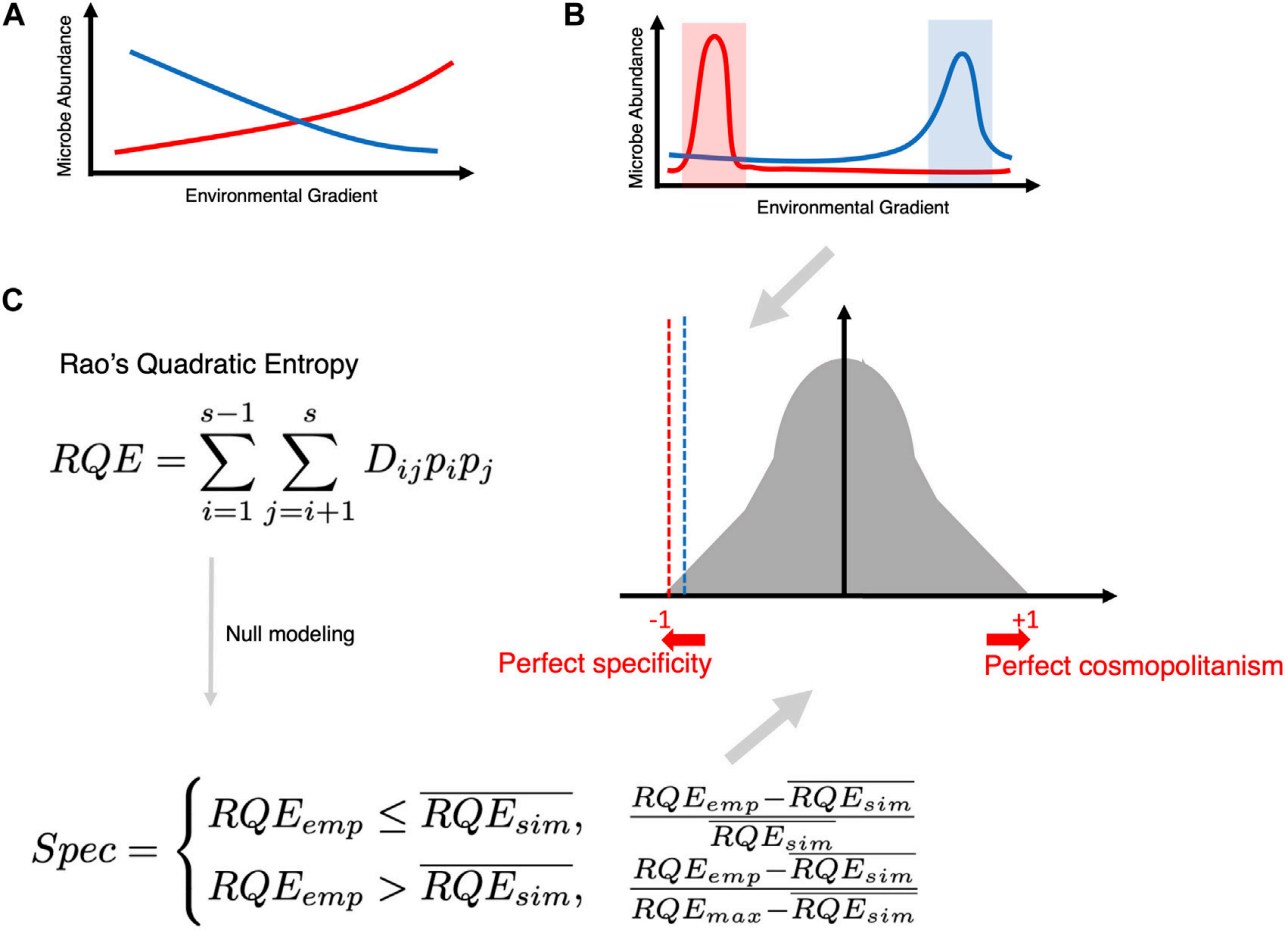
**(A)** Traditional way of finding an association (whether negative or positive) between microbial abundance and a continuous covariate of interest. **(B)** The goal of specificity analysis is to find microbes that become specific within a narrow range of a covariate of interest. **(C)** Creating a null distribution utilizing Rao’s Quadratic Entropy formula (which incorporates the difference of the covariate across two samples i and j as $D_{ij}$ , multiplied by the relative abundance of the microbe across two samples as $p_{i}$ and $p_{j}$ ). These are calculated for all pairwise combinations of samples ( $\frac{s\left(s-1\right)}{2}$ ). The empirical value is then compared with the average of the 999 null distribution values to tag a microbe as specific (Spec ∼ −1) or cosmopolitan (Spec ∼ +1).

Another way of imparting distinction is to tag microbial species as either *specific* (existing within a narrow range of a particular covariate) *or cosmopolitan* (existing in a broader range of a particular covariate). In this regard, R’s Specificity Package (Darcy et al., 2022) is an important development (see Figure 8). It calculates Rao’s Quadratic Entropy (RQE) as $RQE=\sum_{i=1}^{s-1}\sum_{j=i+1}^{s}D_{ij}p_{i}p_{j}$ where microbial abundance $p_{i}p_{j}$ is the multiplication of the abundance of a specific microbe in samples i and j , respectively, each weighted by the difference in the covariate value $D_{ij}$ . A null modeling procedure is then applied (statistical effect size) where 999 random permutations are obtained for the abundance table, and RQE values are then obtained for these random permutations. The deviation of the original RQE from the average of the RQEs of these random permutations then returns a “Spec” number, ranging from −1 to +1, with 0 as the null hypothesis that the genus weights are randomly ordered with respect to sample identity, with perfect *specificity* when Spec approaches −1 and perfect *cosmopolitanism* when Spec approaches +1, and with the null modeling procedure providing additional *p*-values for significance.

**FIGURE 9:**
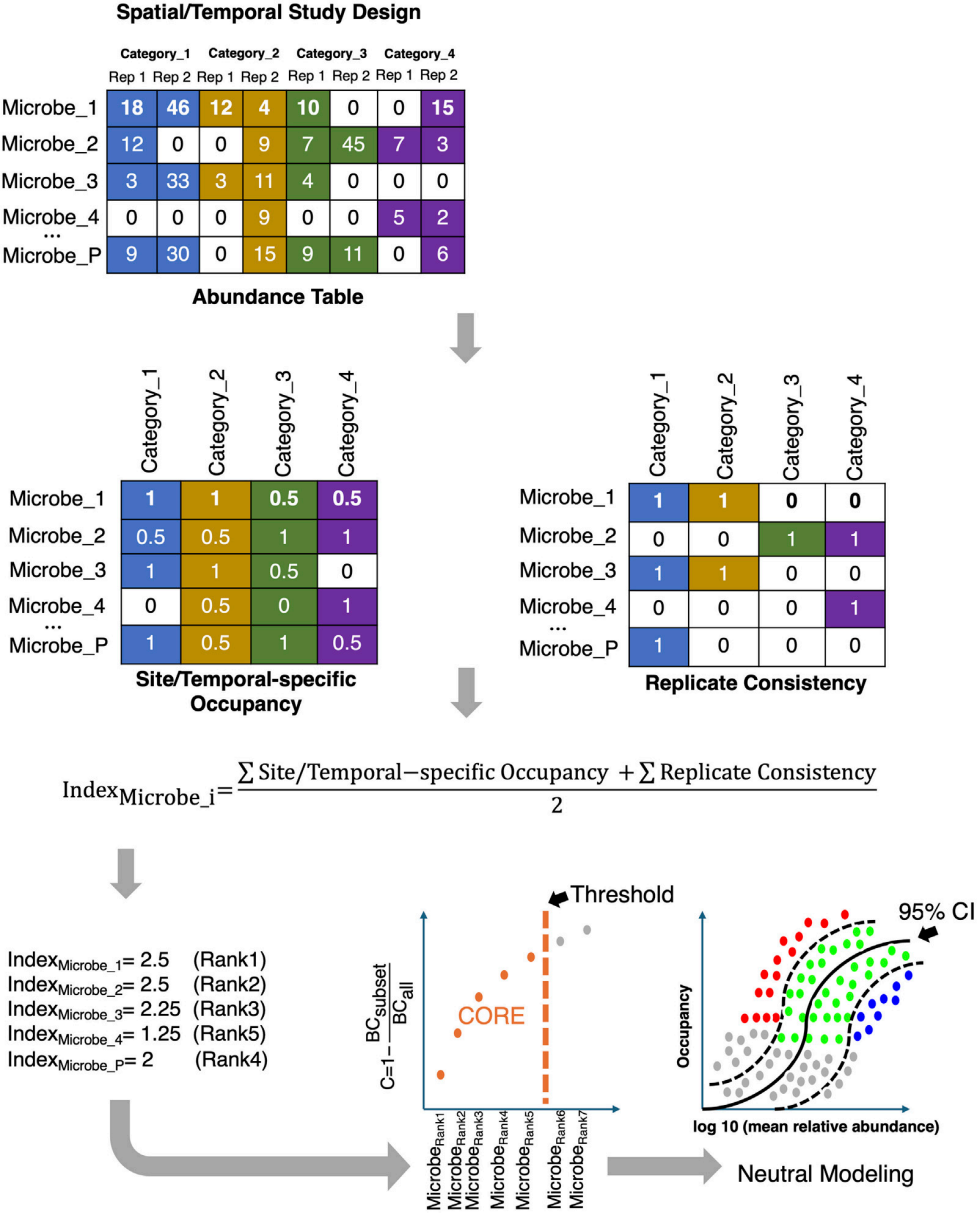
The core microbiome is established by first ranking the microbes based on a combination of two criteria, *Site-specific Occupancy* and *Replicate Consistency*, and then using the Bray-Curtis (BC) contribution of the core subset to the whole community; the core subset is iteratively constructed until the stopping criteria are reached. The core subset is then further discretized to those that: fall above the neutral model (selected by host/environment and represented by red dots in the bottom right figure); fall below the neutral model (selected by dispersal limitation and represented by blue dots); and those that fit the 95% CI of the neutral model (represented by green dots).

Another popular approach to identifying ecological classes is to fit a neutral model (Burns et al., 2016) to the observed microbial abundance-occupancy relationships. This allows the separation of microbial community members into three subsets: a) those that satisfy the 95% confidence interval of the fitted neutral model, and are driven by stochastic processes; b) those that fall above the 95% confidence of the neutral model and are selected by the environment; and c) those that fall below the model, and are driven by the dispersal limitation process (Figure 9).

In summary, the challenges are as follows:

• With the expansion of public databases with deposited sequences, there is a need to understand microbial niche breadth in a meta-analysis setting and to revisit the definition of stability, particularly in light of how niche theory unravels eco-evolutionary processes.
• New metrics need to be developed that identify the ecological roles of microbes for better mechanistic understanding rather than simply identifying microbial species that are up- or downregulated.
• A taxa-centric framework needs to be developed that also incorporates covariates (metadata) into the modeling process based on the hypothesized distinctions.

### Grand challenge: how do you identify a signature microbiome when each ecosystem differs in terms of variability?

One of the less explored areas is the recovery of the core/signature microbiome, which is shared by the majority of samples (or individuals in clinical settings). Traditionally, a core microbiome has been defined as a subset of microbes with high prevalence (typically 50% or 85%) across all samples (Shetty et al., 2017). This prevalence goes down to 30% where core membership is considered stable given individual variabilities (Ainsworth et al., 2015). There is no real consensus on what is an appropriate threshold. Also, different ecosystems have different levels of inter-subject variability. For example, while gut microbial communities may be more similar, vaginal microbial communities show much more variability (Eng and Borenstein, 2018a). Therefore, the challenge is to develop a unified framework where the crisp prevalence threshold is avoided, and the core membership of microbes is dynamically learned from the data. There exists one such recent dynamic strategy (Shade and Stopnisek, 2019) for inferring the core microbiome. The strategy considers (Figure 9) the sample occupancy of microbes at different sites (whether in space or time) along with the replicate information, and then dynamically calculates the minimum occupancy threshold by learning from the data. The ranking of microbes is done using a combination of two metrics: *Site-specific occupancy* (the proportion of microbes within a given site; and *Replicate Consistency* (the consistency of microbes across replicates within a site). After ranking the microbes using the two metrics, the subset of core taxa is constructed by iteratively adding one microbe at a time to the core set of microbes, i.e., from the high-ranked microbes to the low-ranked ones. The contribution of the core subset to beta diversity is then calculated every time a new microbe becomes a member of the core set using the Bray-Curtis contribution, $C=1-\frac{{BC}_{core}}{{BC}_{all}}$ . There are two stopping criteria used in (Shade and Stopnisek, 2019), of which a relaxed criterion for inclusion of a microbe in the core microbiome subset is recommended: *inclusion of* *an additional microbe does not cause more than a 2% increase in the explanatory value by Bray-Curtis distance*. Although not extensively tested on a variety of datasets, this strategy seems promising (in the absence of alternatives) and needs to be explored further including through the development of different metrics to the Bray-Curtis contribution *C*, and other stopping criteria that may be ecologically inspired. The identified core microbiome can then be further regressed against all sources of variation, for example, using the GLLVM framework or can be fitted with a neutral model.

### Grand challenge: how can we integrate additional datasets?

**FIGURE 10:**
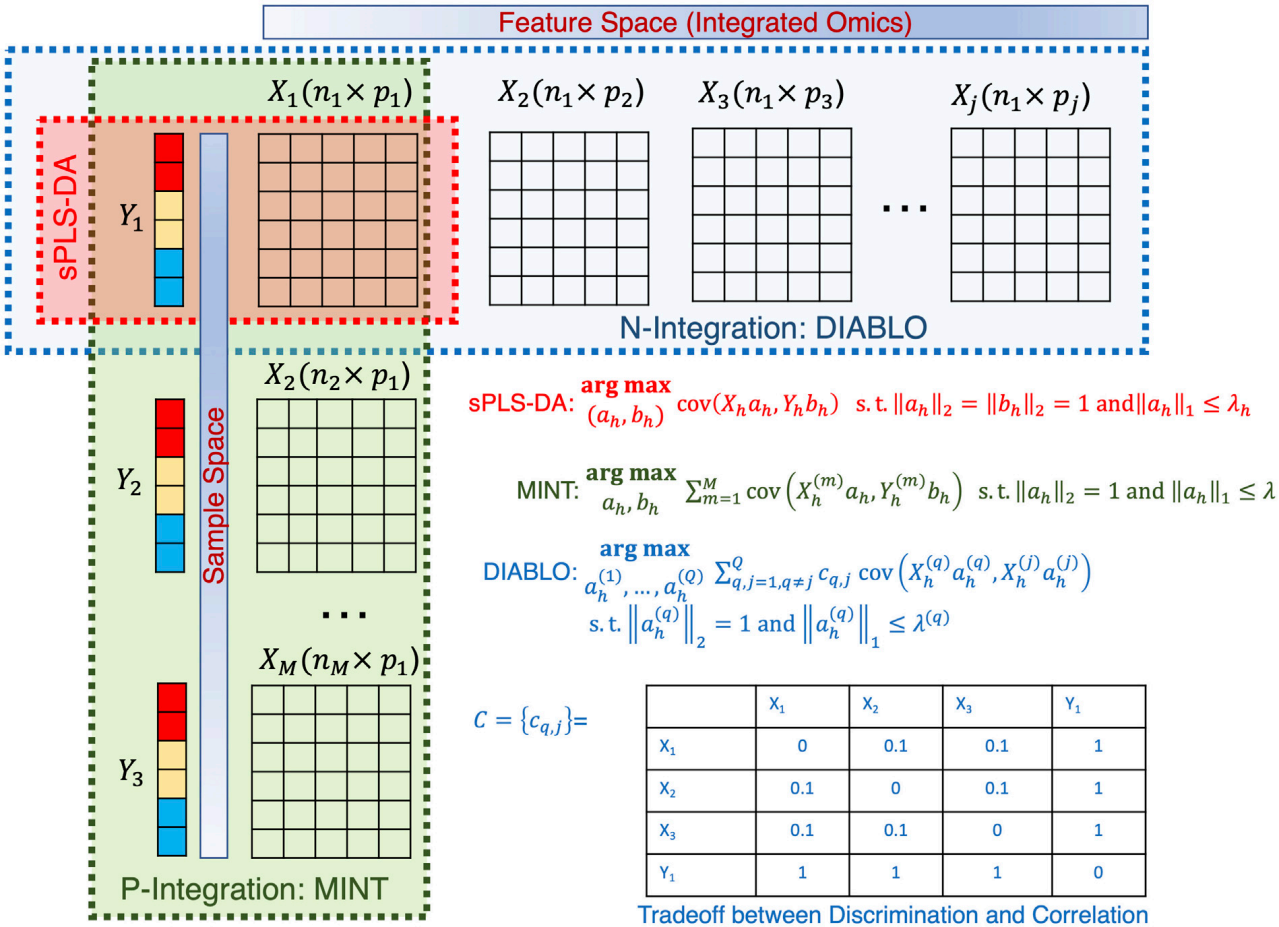
Illustration of the sPLSDA algorithm and its extension. The optimization algorithms are shown for all cases along with the weighting strategy for DIABLO shown in a table.

With the multitude of complex ‘omics datasets that can be attached to the microbiome samples of interest, the process of integrating additional datasets is of great interest. The challenge with ‘omics data is the sheer volume of data (with many features), high noise, sparsity, and potentially missing data points. To overcome these issues and integrate the datasets (often with differing sample numbers), we need to apply tools that reduce the dimensionality of the datasets. One tool commonly used (and variations thereof) is Partial Least-Squares Discriminant Analysis (PLS-DA) (Worley and Powers, 2012). Arguably one of the most crucial developments in microbiome data integration has been the development of ‘mixOmics’ an R package that holds a repository of functions for multivariate analysis of biological data such as dimensionality reduction, and visualization and includes multiple integration tools (Rohart et al., 2017b). This powerful resource allows the integration of microbiome and ‘omics datasets. This package focuses on dimensionality reduction by statistically integrating several datasets using *Projection to Latent Structure* models and their multigroup extensions. The multivariate approaches project the sample matrix X into H latent components giving scores of samples on these components $\left(t_{1},t_{2},\ldots ,t_{H}\right)$ which are defined as a linear combination of the original predictors. The weights of each of the predictors are given by the loading vectors on these components as $\left(a_{1},a_{2},\ldots ,a_{H}\right)$ . The matrix X = $\left(X_{1},X_{2},...,X_{P}\right)$ is then represented in the first latent component as $t_{1}=Xa_{1}=X_{1}a_{1}^{1}+\ldots +X_{P}a_{1}^{P}$ . For each loading vector $a_{h}$ , there is one latent component $t_{H}$ with dimension H≪P . To enable variable selection, the optimization algorithm maximizes the covariance between the scores of two data matrices $X_{i}$ and $X_{j}$ as $cov(t_{h}^{i},t_{h}^{j}$ ) which are subject to LASSO constraints imposed on the loading vectors $a_{h}^{i}$ and $a_{h}^{j}$ . This forces some of the components of the loading vector to go to zero, thus enabling discrimination. The approach is referred to as *sparse Projection to Latent Structure Discriminant Analysis* (sPLSDA). Two of its extensions are widely used: a) the Multivariate INTegrative (MINT) algorithm (Rohart et al., 2017a) called the P-Integration algorithm where the matrices $X_{j}$ share the same features in a multifactorial design; and b)the Data Integration Analysis for Biomarker discovery using Latent cOmponents (DIABLO) algorithm (Singh et al., 2019) called the N-Integration algorithm where the matrices $X_{j}$ originate from multiple modalities each with different features but on the same samples. The optimization strategy is similar to sPLSDA where now the covariance of the scores between multiple matrices is simultaneously optimized either as a simple sum of covariances (MINT) or as a weighted sum of covariances(DIABLO). The weights give DIABLO a trade-off between correlation and discrimination (see Figure 10). There are several challenges associated with the applicability of such approaches:

• The main challenge with these approaches is the appropriate type of normalization model. For datasets where the features are count data, TSS + CLR (Total Sum Scaling followed by Centralized Log Ratio) may suffice. For other types of datasets such as those originating from flow cytometry or metabolomics, there is no real consensus on what is an appropriate normalization measure.
• The P- and N-integration approaches involve optimizing the additive sum of weighted covariances across multiple datasets. Identification of the correct weights that offer reasonable trade-offs between discrimination and correlation is a largely unexplored topic.
• While extensions such as timeOmics (Bodein et al., 2022) have been proposed for temporal datasets that primarily return clustering of time series data across different datasets, the method shows poor performance when there is high inter-replicate variability. Normalization strategies, interpolation strategies (when time points do not match), and pre-processing strategies still need to be further explored.

## Concluding remarks

While there are numerous ways to divulge patterns of interest in microbiome data, and associate them with covariates of interest (metadata), we have discussed those methods that have gained importance in recent years, and this list is by no means exhaustive. Statistical approaches that offer multivariate data integration are few and far between, and those that are used in routine practice have very strong assumptions of linearity. There is room for improvement in these techniques and guided by our experience, we have highlighted the challenges associated with some of these approaches. There is an ever-increasing pressure to utilize analytical techniques that lead to a mechanistic understanding of the ecosystem under study, and perhaps inspired by the work done in ecology, there may be a way. Integration algorithms in recent years have also gained popularity as there is a shift toward incorporating multiple omics technologies in microbiome surveys each offering complementarity. However, we are still far from being able to infer direct causality, although correlation and association inference are well explored. Also, there is a need to develop analytical strategies, that can address nonlinearity, low sample numbers, and unbalanced study designs. Analytical techniques now offer more insight than ever before, but verifying the patterns in the lab or *in situ* is still required to not only yield mechanistic insights, but also to aid in tool development.
